# Strategies for Craniofacial Tissue Engineering: Innovations for Scalable Bone Regeneration

**DOI:** 10.20517/2347-9264.2025.09

**Published:** 2025-06-17

**Authors:** Sofia M. Vignolo, Daniela M. Roth, Lillian Wu, Jameson Cosgrove, Luiz E. Bertassoni

**Affiliations:** 1 Department of Biomedical Engineering, School of Medicine, Oregon Health & Science University (OHSU), Portland, OR 97201, USA.; 2 Knight Cancer Precision Biofabrication Hub, Knight Cancer Institute, OHSU, Portland, OR 97201, USA.; 3 Cancer Early Detection Advanced Research Center (CEDAR), Knight Cancer Institute, OHSU, Portland, OR 97201, USA.; 4 Department of Oral Rehabilitation and Biosciences, School of Dentistry, OHSU, Portland, OR 97201, USA.; 5 Division of Oncological Sciences, School of Medicine, OHSU, Portland, OR 97201, USA

**Keywords:** Bone regeneration, Craniofacial tissue, Tissue engineering, Advanced biomaterials, Translation, Regenerative medicine

## Abstract

Craniofacial tissue engineering offers promising solutions for addressing large bone defects caused by congenital abnormalities, trauma, or disease. Traditional approaches, such as autografts and synthetic materials, are widely used but face limitations, including donor site morbidity, immune rejection, and poor graft integration. Recent advancements in biomaterials, including nanoscale scaffold design, bioceramics, cell-laden hydrogels, and bioactive modifications, present promising strategies to replicate the biological, mechanical, and structural properties of native bone. This review explores innovative strategies to enhance osteoconductivity, osteoinductivity, and osteogenicity of engineered grafts, including the use of advanced biomaterials, immunomodulatory scaffolds, and bioprinting technologies. Key biological challenges are discussed alongside translational barriers. Future directions emphasize the integration of bioprinted, vascularized, multi-phasic tissues, alongside personalized therapies and advanced fabrication techniques, to accelerate clinical adoption. By bridging nanoscale innovations with the demands of large-scale clinical application, this review outlines pathways toward scalable, personalized, and clinically effective solutions to restore functionality and aesthetics in craniofacial reconstruction.

## Graphical Abstract

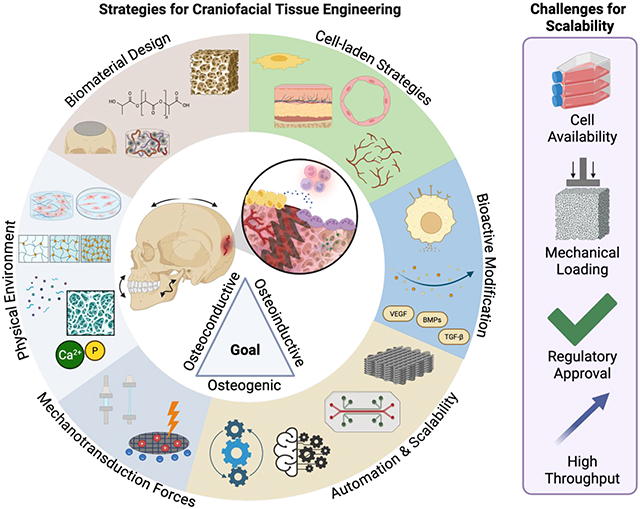

**Strategies and challenges for craniofacial tissue engineering.** Craniofacial bone presents unique challenges for regeneration due to its structural complexity and exposure to dynamic, physiological mechanical loads. Effective tissue engineering strategies aim to replicate native bone, the clinical gold standard, by promoting osteoconductivity, osteoinductivity and osteogenicity. Researchers and clinicians can tailor biomaterial composition, mechanical and physical properties, and bioactive modifications to improve outcomes. The incorporation of cell-laden constructs further enhances regenerative capacity. However, key translational barriers remain, including cell availability, biomaterials enduring mechanical load as the tissue heals, regulatory approval, and scalability challenges in fabrication. Created in BioRender.com.

## 1. INTRODUCTION

Success in craniofacial regeneration requires graft integration with native tissues to restore both function and aesthetics, addressing defects caused by congenital abnormalities, trauma, or disease. Craniofacial defect reconstruction is uniquely challenging due to the diverse tissue types involved and the need to restore functions such as speech, swallowing, breathing, and facial expression. Poor functional integration, suboptimal aesthetic outcomes, or long-term graft failure remain common challenges, underscoring the need for innovative approaches that balance both biological and biomechanical considerations. Bone grafting, with over two million procedures performed annually worldwide, remains the primary solution, but complications such as infection, instability, and inadequate integration can lead to graft failure. Cranioplasty procedures, for instance, report complication rates as high as 25%.^[1–3]^

Given the complexity and mechanical demands of craniofacial bones, advanced tissue engineering strategies are needed to restore morphology and function.^[4]^ Effective grafts must replicate the structural and biological properties of native bone, especially in critical-sized defects that cannot heal spontaneously, while balancing key processes such as inflammation, vascularization, and remodeling with translational requirements like scalability and regulatory hurdles. Emerging innovations in nanoscale scaffold design, cell-laden constructs, and bioprinting offer promising paths forward. This review explores these strategies and their potential to advance scalable, clinically effective solutions for craniofacial reconstruction.

## 2. CURRENT CLINICAL APPROACHES AND LIMITATIONS IN CRANIOFACIAL RECONSTRUCTION

Current clinical standards for craniofacial reconstruction include autografts, allografts, xenografts, biomaterials, biologics and surgical techniques, each with advantages and limitations. Autografts are the gold standard due to low rejection risk and native extracellular matrix (ECM) support, but limited tissue availability and donor site morbidity restrict their use in large defects. Allografts and xenografts are more accessible but carry risks of immune rejection, disease transmission, and lower osteogenic potential.^[5]^ Synthetic materials like calcium phosphate ceramics provide structural support but lack full biological functionality.^[5,6]^ Biologics such as bone morphogenetic protein-2 (BMP-2) and platelet-rich plasma (PRP) have enhanced regenerative outcomes, but require supraphysiological doses to achieve a therapeutic effect, which may cause inflammation or ectopic bone formation.^[7,8]^ Surgical interventions like distraction osteogenesis and Le Fort osteotomies are effective for certain types of defects but involve long recovery times, infection risk, and procedural complexity.^[9–11]^

Building on the limitations of current clinical approaches, recent strategies in craniofacial bone regeneration have shifted toward integrating principles of tissue engineering. Compared to regenerative medicine which harnesses the body’s own repair mechanisms, tissue engineering approaches focus on creating biological substitutes to restore tissue form and function. Emerging techniques include the use of bioactive scaffolds to support osteogenesis and serve as structural templates for cell integration and tissue healing. When combined with stem cells, these scaffolds move beyond traditional regenerative strategies and toward engineered constructs that actively guide bone regeneration. Advances in growth factor delivery, especially with BMP-2, and the use of 3D-printed scaffolds allow for more targeted and efficient bone repair. Additionally, vascularization strategies, including the incorporation of angiogenic and neurovascular cues, are increasingly recognized as essential for graft survival and integration. This review will focus on these tissue engineering-based approaches and their potential to overcome the clinical and biological limitations of traditional grafting methods.

**Figure 1.**
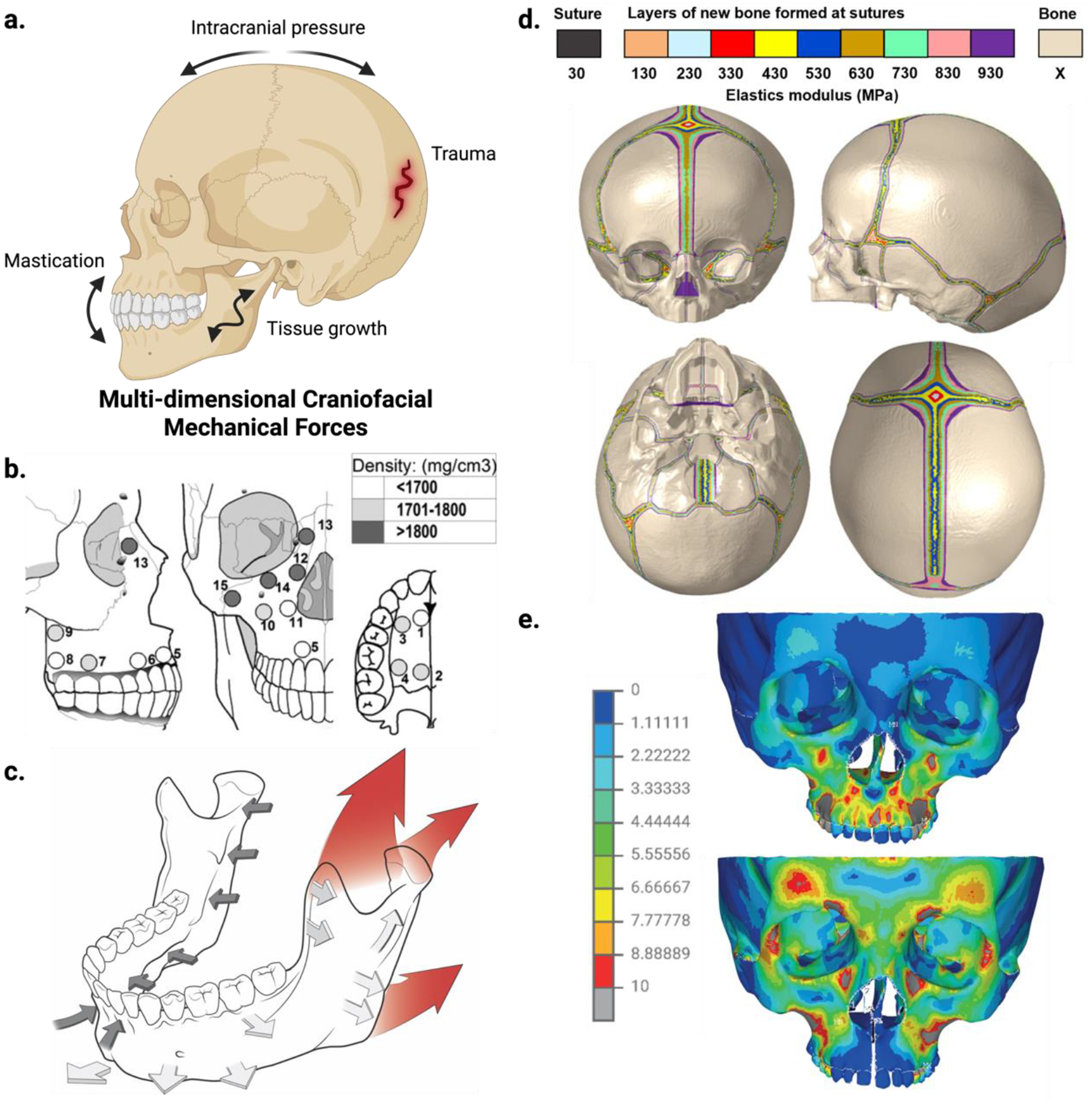
Multidimensional mechanical forces in the craniofacial complex. (a) Key mechanical forces in the craniofacial region include those from mastication, intracranial pressure, trauma, and tissue growth. (b) Regional variations in maxillary thickness and density reflect functional adaptation to these forces. Adapted from Peterson et al., 2006.^[12]^ (c) Pediatric craniofacial bone growth patterns demonstrate dynamic changes in size (red), appositional growth (white), and net resorption (black). Adapted from Farooq 2020.^[13]^ (d) Appositional growth along cranial sutures generates stiffness gradients, as shown in a strain-based in silico analysis at 12 months of age. Adapted from Liang et al., 2024.^[14]^ (e) Finite element modeling reveals altered Huber stress distribution following palatal expansion without osteotomy (top panel) and with sagittal osteotomy (top panel). Adapted from Zawiślak et al., 2020.^[15]^ Created in BioRender.com.

A major hurdle in craniofacial tissue engineering is replicating the mechanical and biological properties of native bone. Unlike long bones, craniofacial bones experience complex, multidirectional forces – including impact, cyclic, and quasi-static loading – that shape their structure and matrix composition (Figure 1^[12–15]^).^[4,16–18]^ To be effective, engineered tissues must mimic both the architecture and biomechanical environment of native bone.

The stability of a bone fracture is heavily influenced by the amount of movement within the fracture gap under physiological loading and can be quantified by the tissue’s elastic modulus, a measure of stiffness.^[19]^ Excessive instability delays healing by exceeding the strain tolerance of repair tissues, leading to proliferation of fibrous or cartilaginous tissue instead of bone formation.^[19]^ Failures in graft integration often stem from high instability at the interface between the graft and native bone, especially when excessive or poorly distributed forces disrupt healing. While some mechanical loading can promote bone regeneration, instability beyond a critical threshold can impair integration and shift the mode of repair.^[20–24]^ The desired outcome of primary bone healing, achieved through intramembranous ossification with minimal callus formation, requires stabilization of the two bone surfaces.^[19]^ In contrast, instability at a bone fracture site typically triggers secondary bone healing through endochondral ossification, characterized by the formation of a cartilage callus intermediate.^[19]^ These biomechanical principles are highly relevant in craniofacial reconstruction, where the intricate anatomy and functional demands of the cranial and facial skeleton necessitate precise control over mechanical loading conditions. Inadequate stabilization can compromise the integration of bone grafts or biomaterial scaffolds, leading to non-unions or suboptimal regenerative outcomes.

Ultimately, successful craniofacial reconstruction requires engineered tissues that replicate bone’s structural and biological properties while withstanding dynamic mechanical loading. Techniques have progressed from traditional grafting approaches, such as autografts and allografts, toward bioinspired strategies grounded in regenerative medicine and design-driven innovations enabled by tissue engineering, including the use of bioactive materials, stem cells, and scaffold-based constructs designed to actively promote bone healing. However, current clinical approaches are often limited by issues such as donor tissue shortages, infection risk, and inadequate integration. These clinical limitations underscore the urgent need for regenerative strategies that integrate mechanical strength, biological function, and anatomical complexity in craniofacial reconstruction. Emerging innovations, including advanced scaffolds, stem cell therapies, and bioprinting, offer promise but face barriers, such as scalability, regulatory hurdles, and challenges in clinical translation. This review explores these complexities and highlights strategies to advance the field toward scalable and effective clinical solutions.

## 3. KEY CHALLENGES IN ENGINEERING BONE GRAFTS TO MIMIC NATIVE BONE

Replicating the biological, mechanical, and structural properties of native bone remains a central challenge in craniofacial tissue engineering. Bioinspired bone grafts aim to support vascularization, bone bridging, and functional integration by leveraging the intrinsic biology of bone healing.^[25,26]^Achieving these outcomes requires addressing interrelated biological processes - namely inflammation, vascularization, and remodeling, while also overcoming translational barriers such as scalability and regulatory complexity.^[26]^ Advances in fabrication technologies and material design offer opportunities to develop next-generation grafts that restore function and improve patient outcomes.

### 3.1. Biological Challenges

Successful bone regeneration depends on restoring homeostasis through tightly coordinated processes that include, but are not limited to, inflammation, vascularization, and remodeling.^[27,28]^ Disruption or dysregulation of any phase can impair healing and compromise graft integration. Inflammation is essential for initiating repair but must be rightly regulated to prevent chronic immune responses that interfere with vascular development or osteogenesis. Similarly, vascularization not only delivers oxygen and nutrients but also drives regenerative signaling and supports tissue remodeling. Another critical factor is scaffold degradation. Materials must degrade at a rate that supports new bone formation and the gradual development of mature bone, providing temporary structural support without compromising mechanical stability or long-term integration. This interplay between biological processes and material behavior underscores the need for grafts that harmonize with the body’s natural healing dynamics. Engineering constructs that can engage these mechanisms effectively remains a key benchmark for clinical success.

#### 3.1.1. Initial Inflammatory Phase

The addition of engineered constructs into the physiological system to facilitate or accelerate bone healing presents challenges to this finely tuned process, particularly in managing the inflammatory response during the initial stages of healing.^[28]^ This phase, characterized by immune cell recruitment to clear debris and stimulate mesenchymal stem cell (MSC) migration, is critical for initiating regeneration and is followed by osteoblast-driven bone formation as inflammation subsides.^[28,29]^ However, prolonged or poorly regulated inflammation can result in chronic inflammation that impairs healing and disrupts graft integration.^[30]^ Likewise, insufficient clearance of necrotic tissue or surgical debris can prolong inflammation, creating an unstable microenvironment that disrupts tissue repair and increases the risk of graft failure.^[31,32]^ An excessive immune response can lead to fibrous capsule formation, as debris becomes sequestered within the tissue, further impeding repair.^[31]^ Chronic inflammatory conditions, including autoimmune disorders, exemplify how dysregulated immune activity can impair healing by promoting fibrosis and scarring, thereby prolonging recovery.^[33]^

Inflammation and bone regeneration are paradoxically linked: while excessive inflammation is detrimental, specific pro-inflammatory mediators are indispensable for initiating healing.^[28,34]^ The immune system plays a significant role in bone repair and osteogenesis, and nonspecific immunosuppression may compromise healing capacity.^[35]^ For example, elevated levels of myeloid-derived suppressor cells (MDSCs) and the immunosuppressive cytokine interleukin (IL)-10 have been associated with delayed or incomplete bone regeneration, underscoring the need for a balanced inflammatory response.^[34]^ Immune cells and their secreted factors also influence angiogenesis by releasing pro-angiogenic signals such as vascular endothelial growth factor (VEGF), a key regulator of blood vessel formation.^[36]^

Importantly, biomaterials themselves play an active role in modulating the immune response. Material composition, stiffness, surface topography, and degradation products can influence macrophage polarization toward either a pro-inflammatory (M1) or pro-regenerative (M2) phenotype. Materials that promote M2 polarization, such as certain bioactive ceramics or natural polymers, have been associated with enhanced vascularization, MSC recruitment, and osteogenesis. Conversely, materials that trigger sustained M1 activity may impair healing by prolonging inflammation and limiting graft integration. This interplay between the immune and vascular systems underscores their collective importance in bone regeneration and highlights the need for precise modulation of inflammatory activity to support successful graft integration.^[36]^

#### 3.1.2. Vascularization Phase

Vascularization remains one of the most significant challenges in tissue engineering, particularly for large constructs where nutrient diffusion limits cell viability and integration.^[37]^ Both angiogenesis, the sprouting of new vessels from existing vasculature, and vasculogenesis, de novo vessel formation from endothelial progenitor cells, are essential for bone repair. These processes supply oxygen and nutrients to the regenerating tissue and enable immune cell infiltration, supporting healing and remodeling.^[36]^ Animal studies show that enhanced angiogenesis accelerates bone repair, whereas insufficient vascular perfusion impairs fracture healing.^[36,38]^

Interestingly, angiogenesis and osteogenesis appear uncoupled in calvarial bone regeneration, in contrast to long bones.^[39]^ In calvarial defects, vascular sprouts first establish a network within the lesion, followed by invasion of osteoprogenitor cells from the periosteum to drive ossification. Conversely, femoral fractures exhibit simultaneous angiogenic and osteogenic activity. These differences highlight the need for bone-specific vascularization strategies.

To avoid avascular necrosis, particularly at the construct core, tissue-engineered grafts are typically designed to remain within ~200μm of capillaries – the diffusion limit for oxygen and nutrients.^[6,17,40]^ Beyond proximity, the type of vasculature formed also affects bone healing.^[41]^ For instance, type H vessels are closely associated with osteogenic activity and are implicated in bone development and repair.^[41,42]^ Vascular branching patterns and architecture, shaped by the local microenvironment, further influence construct integration and remodeling.^[43]^

Vasculature and associated perivascular cells also function as key sources of pro-osteogenic growth factors, contributing to a regenerative endocrine loop.^[44]^ Blood vessels release angiocrine signals that promote osteogenesis and recruit osteoprogenitor cells.^[41]^ In turn, osteoblasts and other bone cells regulate angiogenesis to meet the metabolic demands of repair.^[41,45]^ This reciprocal crosstalk is shaped by local signaling cues – an area of engineering that remains underexplored.^[41,46]^ To fully restore the native bone function, newly formed vasculature must integrate into a calcified matrix with bone marrow cavities to support hematopoietic and metabolic functions.^[38]^ Establishing this vascularized and structurally mature microenvironment is critical for long-term remodeling and functional integration of the graft.^[47]^

#### 3.1.3. Remodeling Phase

**Figure 2.**
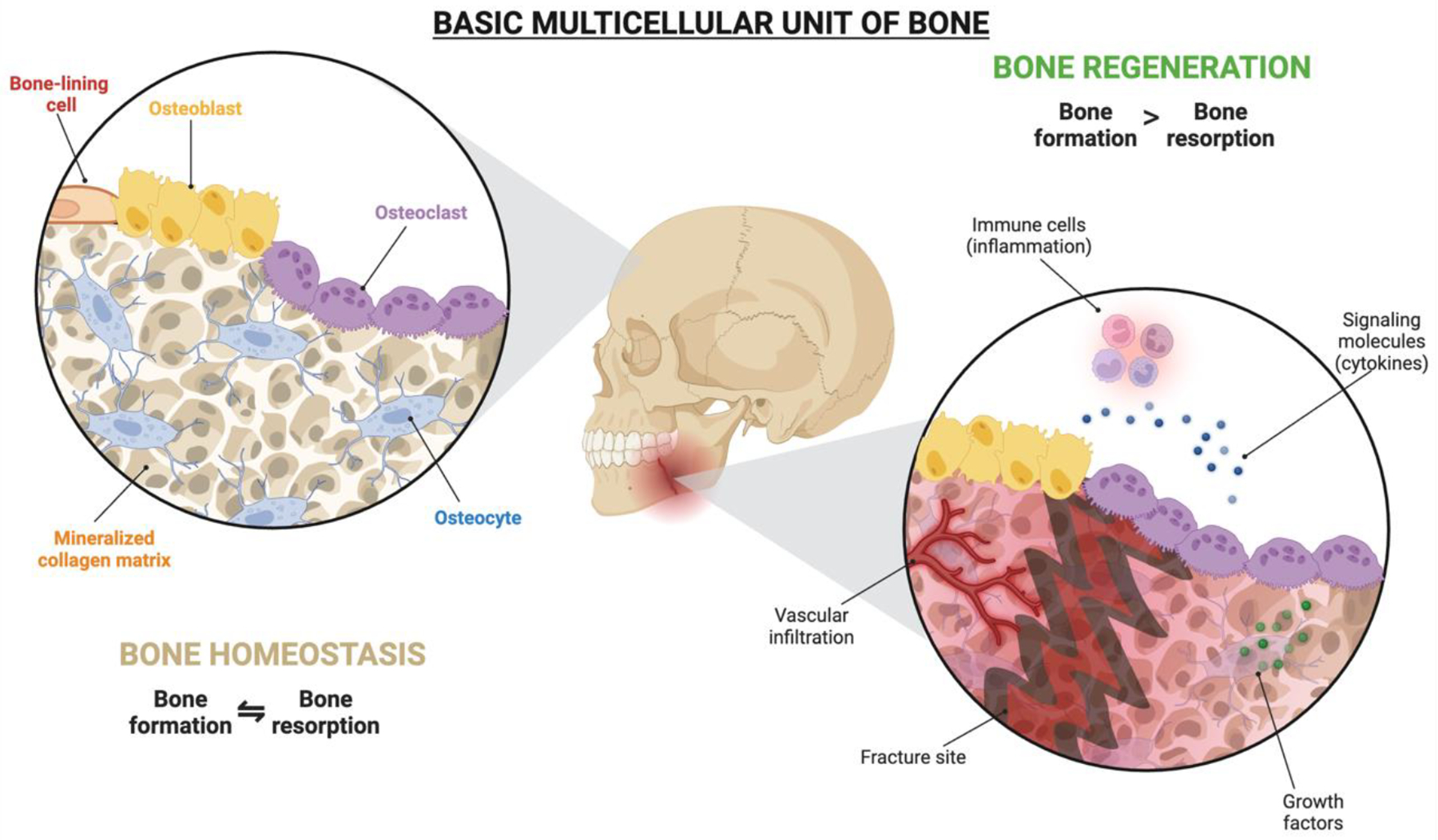
The basic multicellular unit (BMU) of bone. The BMU represents the coordinated activity of bone-lining cells, osteoblasts, osteoclasts, and osteocytes on a mineralized collagen matrix, maintaining bone homeostasis through balanced bone formation and resorption. In contrast, bone regeneration involves the activation of immune cells, signaling molecules, and growth factors at the fracture site. This process is marked by vascular infiltration and increased new bone formation to restore structural integrity and function. Created in BioRender.com.

Following vascularization, successful bone regeneration requires effective remodeling and osseointegration, transforming the graft into functional, load-bearing tissue.^[46]^ Remodeling refers to the continuous renewal and adaptation of bone, involving the removal of old bone and its replacement with new tissue that is structurally refined and functionally optimized.^[48]^ Osseointegration establishes a direct, load-bearing connection between bone and graft material.^[49]^ In resorbable materials, osseointegration involves gradual material degradation coupled with new bone formation, while in non-resorbable materials, it relies on maintaining a permanent, stable interface with the surrounding bone.^[50]^ These processes are driven by dynamic interactions among various bone-resident cell lineages. Osteoblasts (bone-forming cells) from the mesenchymal lineage and osteoclasts (bone-resorbing cells) from the hematopoietic lineage work in coordination to remodel bone tissue. Initially, remodeling was understood as the reciprocal interaction between osteoclasts and osteoblasts.^[51,52]^ However, studies continue to reveal that all bone cells, including progenitors, contribute simultaneously and at various stages of differentiation to form the basic multicellular unit responsible for remodeling (Figure 2).^[53,54]^ This active, coordinated cellular activity influences bone renewal and structural adaptation, emphasizing the need for engineered constructs to support these intricate physiological processes.

Together, inflammation, vascularization, and remodeling form an interdependent triad that must be precisely modulated to achieve successful integration and long-term functionality of engineered bone grafts. These interconnected processes remain central challenges in the field and a major focus of ongoing research aimed at improving craniofacial regeneration outcomes.

The ideal bone graft material should closely replicate the mechanical properties of native tissue and degrade at a rate synchronized with tissue growth, ensuring the implanted material to be gradually replaced by newly formed bone.^[55–57]^ Additionally, the material's degradation behavior should account for the direct influence of mechanical loading, supporting the temporal needs of the bone regenerative process.^[58]^ If the graft degrades too quickly, it may fail to provide adequate physical support for the newly forming bone, compromising the healing process. Conversely, if the graft degrades too slowly, it can hinder the critical remodeling process, preventing proper integration with the host tissue.^[59]^ However, this concept remains debated, as some propose that scaffolds should primarily act as space maintainers, facilitating bone ingrowth and maturation, which may not always coincide with the rate of bone deposition. Therefore, achieving the right balance in graft degradation and bone formation rates is essential for ensuring long-term success in bone regeneration.

Aligned with physiological processes, the engineering of innovative bone grafts depends on balancing interconnected biological processes including inflammation, vascularization, and remodeling, all of which dynamically influence and regulate one another. Inflammation, while critical for initiating healing, must be carefully regulated to avoid interference with vascular development or remodeling. Vascularization, essential for nutrient delivery and osteogenesis, also supports remodeling by driving regenerative signaling within the graft. Additionally, the degradation rate of the graft scaffold requires precise synchronization with bone formation, providing structural support without hindering integration. Advancing bone regeneration requires pushing beyond current clinical capabilities, urging clinicians to recognize the interplay of these factors and inspiring researchers to design grafts that integrate with the body’s natural healing mechanisms, setting a new standard for successful outcomes.

### 3.2. Translational Challenges

Translational challenges in bone tissue engineering often begin at the discovery phase, where preclinical models fail to replicate key aspects of human regeneration.^[60]^ Rodents are commonly used for basic science research, while large animals like pigs, sheep, and goats are employed for bone defect studies.^[61]^However, these models frequently diverge from human biology. Humanized systems, including patient-derived xenografts, organ-on-a-chip systems, and bioprinted cell-laden constructs, offer more clinically relevant alternatives to study scaffold integration, immune responses, and vascularization in vitro.^[62–67]^

A major hurdle in clinical translation is the scalability of fabrication methods.^[68]^ While nanoscale scaffolds can mimic ECM features and direct cellular behavior, translating these innovations to clinically sized craniofacial grafts remains a challenge.^[69,70]^ Craniofacial bones add further complexity due to their heterogeneous morphology - characterized by curved and ridged surfaces, varying cortical and trabecular thickness, muscle attachment sites, and neurovascular channels.^[12]^ These anatomical intricacies demand advanced, customizable scaffold designs beyond uniform or repetitive structures.^[71]^

Implementation also raises questions regarding regenerative cell sources and their compatibility with the functional and developmental properties of the defect site.^[72,73]^ Craniofacial bones develop via both intramembranous and endochondral ossification. For instance, most calvarial bones form directly from MSCs, while the cranial base and parts of the mandible follow an endochondral ossification pathway through a cartilage intermediate.^[74]^ As many nanoscale scaffolds are optimized for promoting direct MSC-to-osteoblast differentiation, their suitability for endochondral repair is unclear.^[75]^ Recent advances in biomimetic scaffold design are beginning to address this gap.^[40]^

Further complexity arises from differences in embryonic origin.^[76,77]^ Frontal bones (derived from neural crest cell) and parietal bones (from paraxial mesoderm) differ in signaling profiles, affecting their regenerative behavior. Frontal bones show greater BMP, FGF, and Wnt activity, while parietal bones exhibit stronger TGFβ signaling and greater susceptibility to apoptosis.^[78]^ These suggest that a one-size-fits-all approach may be insufficient for craniofacial bone engineering, reinforcing the need for methods that accommodate the anatomical and developmental complexity of the craniofacial complex.

In addition to developmental differences, successful craniofacial reconstruction requires integrating diverse bone types, such as cortical and cancellous bone, which differ in mechanical strength and regenerative capacity. Cortical grafts provide structural support, while cancellous grafts promote faster revascularization due to their porosity, and their combined use can optimize outcomes.^[79,80]^ However, current techniques often apply uniform strategies across anatomically distinct regions, limiting integration and repair in sites with complex geometry or load-bearing demands. These challenges highlight the need for grafts and surgical approaches tailored to the structural and biological nuances of each craniofacial defect.

Clinical adoption introduces further barriers. New biomaterials require not only regulatory approval and validated manufacturing pipelines, but also surgical training in handling, storage, and application. Implementation science offers strategies to integrate new technologies into clinical workflows through standardization and guideline development.^[81]^ Regulatory barriers further slow adoption, as engineered bone constructs must meet stringent safety and efficacy standards.^[72]^ These barriers – scaling, cost, regulation, and clinical training – must be addressed to successfully translate bone tissue engineering from the lab to patient care.^[82]^ Addressing anatomical variability, developmental origins, and clinical workflow requirements is essential for translating next-generation bone scaffolds into effective, scalable craniofacial therapies.

## 4. BIOMATERIALS FOR TISSUE ENGINEERING

To address the current limitations in craniofacial tissue engineering, researchers are advancing biomaterials that improve functionality, scalability, and clinical applicability. A major focus is on nanoscale scaffold design, which mimics the extracellular environment to support cell attachment, signaling, and growth. Biomaterials such as bioceramics, synthetic polymers, and organic polymers, and metals are widely used for their various ability to emulate aspects of the native bone microenvironment (Table 1).

Mimicking bone at the nanostructural, compositional, and mechanical levels enhances integration and promotes biologically functional regeneration. Scaffold design parameters - including pore size^[132,133]^, geometry^[134]^, fiber alignment^[135]^, stiffness^[136]^, immunomodulatory effects^[137]^, and cytokine signaling^[138]^ – must be carefully tuned. The success of bone scaffolds depends on their osteoconductivity (supporting cell attachment and bone matrix formation), osteoinductivity (recruiting and inducing progenitors into bone-forming cells), and osteogenicity (stimulating new bone formation and maturation).^[139]^ While each material class offers distinct advantages, combinations and modifications are often required to achieve optimal regenerative outcomes.

### 4.1. Bioceramics

Bioceramics, including calcium phosphates (CaPs)^[140]^, such as hydroxyapatite (HA)^[141]^ and β-tricalcium phosphate (β-TCP)^[132,142]^, calcium silicates (CS)^[143]^, and bioactive glasses (BGs),^[144]^ mimic the mineral phase through ionic and covalent bonding.^[145,146]^ HA constitutes ~65% of native bone’s inorganic mass, while β-TCP is more rapidly resorbed, supporting remodeling.^[147,148]^ These materials offer excellent biocompatibility and osteoconductivity, but their brittleness and porosity limit mechanical strength.^[149]^ Porosity is necessary for cell infiltration and vascularization but weakens structural integrity. Bioceramics are available as injectable cements, particulate granules, or 3D-printed forms, with commercial examples including BoneSource^™^, α-BSM^®^, Biocement D^®^, Mimix^™^, and Cerasorb^®^.^[109,150]^

Bioceramics degrade into bioresorbable end-products, some of which also possess bioactive properties that can promote tissue regeneration. For example, the degradation of bioactive glass begins with the dissolution of the silica, which promotes the formation of a HA layer on the material’s surface. As a result, the bioactive HA layer continues to grow as the original glass material degrades, and byproducts Na^+^, Ca^2+^ ions, and silicic acid are released.^[92]^ Similarly, calcium silicates experience hydrolysis and ion exchange with interstitial fluid, forming a HA surface layer and releasing silicon ions that can up-regulate osteoblast proliferation, differentiation and bone-related gene expression.^[89]^ Calcium phosphates are degraded through a combination of passive solubilization and macrophage and osteoblast phagocytosis, where the end products of calcium and phosphate ions are metabolized for bone formation or naturally eliminated.^[86]^ Recent advancements among bioceramics in craniofacial regeneration have focused on optimizing biomaterial composites for maximized osteogenic potential and combining these materials with biofabrication techniques such as additive manufacturing.^[151]^

### 4.2. Synthetic Polymers

Common synthetic polymers include polycaprolactone (PCL)^[152]^, polylactic acid (PLA)^[153]^, polyglycolic acid (PGA)^[154]^, and polyethylene glycol (PEG)^[155]^, which are widely used due to their mechanical tunability, FDA approval, and compatibility with scalable manufacturing.^[156]^ Copolymers like PLGA allow adjustable degradation via monomer ratio.^[157,158]^ Hydrophobicity also limits protein adsorption and cell adhesion.^[159]^ Surface modifications, such as plasma treatment, ECM coatings, or bioactive molecule incorporation, are used to improve bioactivity. PCL, in particular, has broad use in cranioplasty^[160,161]^, orbital floor repair^[162]^, nasal and maxillofacial reconstruction^[163,164]^, and intraoral defects^[165]^. Its advantages include flexibility and long-term mechanical stability, but slow degradation (3–4 years) and acidic byproducts can cause wound dehiscence or infection in some cases.^[102,166]^

When implanted, aliphatic polyesters such as PCL, PLA, PGA, and their copolymers are degraded through hydrolysis of their ester backbone, which releases acidic monomers and oligomers. Although these products are bioresorbable and degrade into endogenous metabolites, their accumulation pose risk of localized acidity and inflammation particularly in poorly vascularized sites, which in severe cases can lead to metabolic acidosis.^[167]^ While PEG alone is not biodegradable, it is often modified to be degradable.^[100,104]^ For example, poly(ethylene glycol) diacrylate (PEGDA), a common derivative of PEG, is hydrolyzed at its ester linkages and oxidized at its acrylate end groups, such that resulting PEG fragments and acrylates are small enough for renal clearance.^[106]^ Synthetic polymer scaffolds have recently been engineered to incorporate drug or growth factor loading, and to be compatible with advanced biofabrication techniques.^[168,169]^

### 4.3. Natural Polymers

Natural polymers such as collagen^[170]^, gelatin^[171]^, chitosan^[172]^, and silk^[173]^ provide biocompatibility and low immunogenicity. Collagen, the primary organic component of bone, supports osteogenesis due to its native cell-binding domains.^[174]^ However, these materials often lack mechanical strength and degrade rapidly, limiting use in load-bearing applications. Composite strategies – e.g., mineralized microgels combining collagen and calcium phosphate – enhance osteoconductivity and ECM mimicry.^[156,175]^ Demineralized bone matrix (DBM), derived from decalcified allograft bone, retains embedded growth factors like BMPs, supporting osteoinduction.^[110]^ DBM’s limitations include donor variability and residual disease transmission risk.^[111,176]^ Alginate and chitosan are also explored for their gel-forming ability and antibacterial properties but are typically used with other materials due to mechanical limitations. Popular DMB-based clinical products include Grafton^®^, Regenafil^®^, and Dynagraft^®^.^[109,177]^

The degradation of collagen, DMB, and gelatin is facilitated by metalloproteinases such as collagenase, leaving bioresorbable end-products of amino acids, collagen fragments, and peptides. Silk is degraded by host immune cells and proteases into amino acids glycine and alanine.^[104,114,120,178]^ Additionally, its degradation rates can be modulated based on the amount of β-sheet and its secondary structure. Similarly, chitosan degradation is mitigated by lysozymes and lipase into amino acids and saccharides.^[118]^ As humans lack the enzyme alginase, pure alginate is non-biodegradable. However, most applications of alginate in tissue engineering ionically cross-link or modify the material to enable degradation.^[104,179]^ Similar to synthetic polymers, recent research among natural polymers has focused on composite materials to improve osteogenicity and modulate degradation rates as well as the incorporation of therapeutics.^[180]^

### 4.4. Metals

Although metals do not inherently induce new bone formation, they continue to be a widely used biomaterial in craniofacial reconstruction for their mechanical strength, biocompatibility, and corrosion resistance.^[181]^ In particular, titanium meshes and plates are FDA-approved for use in cranioplasty^[182]^, alveolar ridge repair^[183]^, and mandibular reconstruction.^[184]^ Titanium’s formability allows for patient-specific shaping, while its passive oxide layer minimizes corrosion and cytotoxicity.^[185,186]^ However, the stress-shielding effect, where material stiffness reduces load-transfer to bone, can cause resorption and implant loosening. Metal ion release from wear or corrosion may also trigger inflammatory responses or peri-implantitis.^[187,188]^ Other examples of metal-based biomaterials that have been used in craniofacial surgery include biodegradable magnesium fracture fixation screws MAGNEZIX^®^^[189,190]^, magnesium bone void filler OsteoCrete^®^^[131]^, Luhr^®^ Modular Craniomaxillofacial/Mandibular Vitallium^®^ Chromium-Cobalt Fixation System^[191]^, and Zinc-doped bone substitute Sil-Oss^®^^[127,192]^.

Although metals such as titanium, stainless steel, and chromium-cobalt are not inherently biodegradable, they undergo electrochemical corrosion when in contact with interstitial fluid and experience general wear overtime. Controlling corrosion remains crucial, as excessive ions in the body can trigger immune rejection and disrupt ion regulated enzymes and proteins.^[130,193]^ Biodegradable, bioresorbable metals such as magnesium, zinc, iron, and their alloys have emerged as a promising solution, as their corrosion products are naturally metabolized ions which minimize the risk of toxicity. Additionally, they offer superior mechanical strength to other biodegradable biomaterials.

Other recent advancements within the field have sought to improve osseointegration and reduce immune reactions by exploring porous metals like tantalum^[194–197]^, surface nano-coatings^[198]^, roughening^[199]^, or bioactive functionalization^[200]^. Though most metals are non-degradable, they remain essential for hybrid implants where high mechanical strength is required. Selecting and optimizing biomaterials remains foundational to craniofacial tissue engineering, as no single material fulfills all structural and biological requirements. Continued innovation in composite scaffolds and bioactive modifications is essential to advance toward clinically effective, personalized solutions.

## 5. INNOVATIVE STRATEGIES FOR TISSUE ENGINEERING

**Figure 3.**
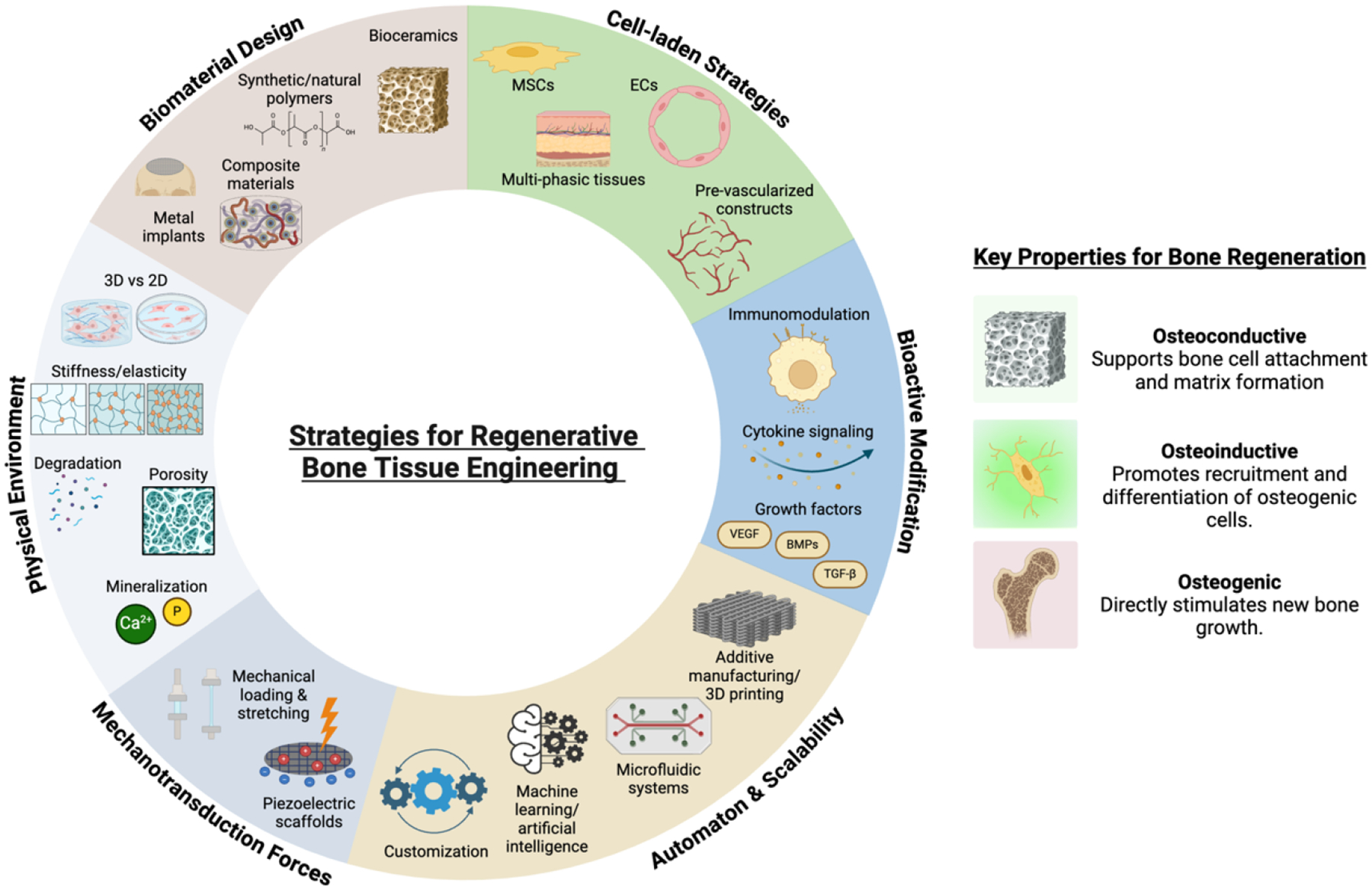
Overview of strategies for regenerative bone tissue engineering. The diagram highlights key approaches to achieve the essential properties for bone regeneration—osteoconductivity, osteoinductivity, and osteogenicity. *Biomaterial design* relates to the materials used such as bioceramics (e.g., hydroxyapatite), synthetic/natural polymers (e.g., PLA and collagen), composite materials, and metals for mechanical strength and structure to support regeneration. *Cell-laden strategies* leverage MSCs and endothelial cells (ECs) through their delivery via scaffolds, microgels, or hydrogels to generate pre-vascularized constructs supporting angiogenesis. *Bioactive modifications* utilize immunomodulation, cytokine signaling, and growth factors like VEGF, BMPs, and TGF-β to support bone repair. *Physical microenvironmental factors*, including dimensionality (i.e. 2D vs 3D), stiffness, elasticity, porosity, degradability, and mineralization, are critical for creating biologically relevant scaffolds. *Mechanotransduction forces*, such as cyclic loading and piezoelectric scaffolds, mimic physiological bone stresses. *Automation and scalability*, through tools like bioprinting, microfluidics, and artificial intelligence, enable the efficient production of clinically relevant constructs. Created in BioRender.com.

The efficacy of regenerative bone scaffolds depends on three key properties: osteoconductivity (supporting cell attachment and bone matrix formation), osteoinductivity (recruiting and inducing osteogenic cells to adopt a mineralizing phenotype), and osteogenicity (stimulating new bone growth and the formation of mature bone).^[139]^ While each biomaterial possesses unique inherent properties that are ideal for craniofacial regeneration, they are rarely applied exclusively.^[201]^ The modification and optimization of these materials remain crucial to maximizing their osteoinductive, osteoconductive, and osteogenic potential (Figure 3).

### 5.1. Strategies to Improve Osteoconductivity

Improving osteoconductivity enhances scaffold support for cell attachment, migration, and matrix deposition. Surface modifications, such as hydroxyapatite coating, increase osteoblast activity.^[202]^ Pore geometry also influences integration: smaller pores promote angiogenesis, while larger pores enable cell migration.^[203]^ Techniques like porogen leaching^[204]^ and electrospinning^[205–207]^ generate porous or nanofiber-based ECM-mimicking scaffolds. For instance, electrospun P34HB fibers support MSC adhesion and calvarial bone regeneration.^[208]^ A widely used biochemical strategy is the functionalization of scaffolds with short peptide sequences such as RGD (Arg-Gly-Asp), which mimics cell adhesion motifs found in fibronectin and other ECM proteins. RGD modification enhances integrin-mediated cell attachment and spreading, improving cellular responses on both natural and synthetic scaffolds. Incorporating RGD into hydrogels, electrospun fibers, or 3D-printed constructs has been shown to promote osteogenic differentiation and improve scaffold integration.^[209–212]^ In addition, incorporating bioactive molecules (e.g., collagen^[213]^, HA^[214,215]^, manganese^[216]^) or creating composite scaffolds^[217,218]^ can further enhance performance by combining the mechanical or biological advantages of multiple materials.

### 5.2. Strategies to Improve Osteoinductivity

Osteoinductive scaffolds guide osteoprogenitor cells toward bone-forming phenotypes, often by mimicking the natural bone healing milieu rich in growth factors like BMP-2, PDGF, TGF-β, IGFs, PRF, and VEGF.^[46,109,213,219]^ These factors can be delivered using nanoparticles or hydrogels that allow for controlled, sustained release and protection from rapid degradation. Material stiffness is another critical parameter influencing osteoinductivity.^[220]^ Substrates with tunable stiffness can mimic the progressive changes in mechanical cues during bone healing and direct stem cell fate through mechanotransduction pathways. Inorganic ions such as calcium (Ca^2+^) and phosphate (PO_4_^3-^) can also modulate the osteoinductive potential of scaffolds.^[221,222]^ These ions are essential components of bone mineral and act as biochemical cues that promote osteogenic gene expression and matrix mineralization.^[223]^ Scaffold materials that release Ca^2+^ and PO_4_^3-^, such as calcium phosphate ceramics, bioactive glasses, or mineralized collagen, can create a microenvironment favorable for bone regeneration. Bioactive peptides and functionalized nanoparticles can further improve spatial delivery and osteogenic signaling, with HA and TGF-β1 frequently used to promote bone formation.^[147,224–226]^

### 5.3. Strategies to Improve Osteogenicity

Enhancing osteogenicity involves promoting bone matrix deposition through scaffold-cell systems. Incorporating osteogenic cells (e.g., MSCs, pre-differentiated osteoprogenitors) can accelerate bone formation.^[227]^ Mechanical loading – including cyclic stretching - upregulates RUNX2 and BMP-2 and improves *in vivo* regeneration.^[228,229]^ Piezoelectric materials^[230,231]^, like barium titanate (BaTiO_3_)^[232]^, convert mechanical forces into electrochemical signals that stimulate ECM mineralization and macrophage polarization^[233–235]^. Direct electrical stimulation also shows potential to enhance osteogenic gene expression.

### 5.4. Strategies for Engineering Cell-Laden Scaffolds

To further recapitulate the tissue repair microenvironment, the incorporation of cells into scaffolds, referred to as “cell-laden scaffolds,” has become a transformative approach in tissue engineering, particularly when utilizing stem cells. These cells have proven effective in facilitating bone regeneration by secreting osteogenic and osteoconductive growth factors, recruiting host immune cells, stimulating angiogenesis, and supporting the structural and mechanical integrity of the ECM.^[236,237]^ Historically, organ models and tissue engineering scaffolds have been two-dimensional, where cells were cultured on flat, planar surfaces. Even when scaffolds are structured in 3D, seeding cells on these surfaces still provides a 2D environment for cell attachment, which is different from the 3D fully embedded structure that cells naturally experience in native bone. These systems lack the structure to support complex intercellular and cell-ECM interactions found *in vivo* and alter cell morphology, which can impact function and biomarker expression.^[238]^ Thus, the field has shifted towards 3D culture environments instead in an effort to better recapitulate the native cell microenvironment. 3D cultures not only encourage more biologically accurate cell differentiation, proliferation, function, and response to stimuli, but also offer a more realistic representation of oxygen, nutrients, and cytokine diffusion.^[239]^ By translating these concepts to bone regeneration, a 3D scaffold can stimulate greater host cell infiltration, effective biomaterial integration, allow for multi-cell interactions, and simulate native bone mechanotransduction forces.^[240]^ Ultimately, 3D scaffolds offer a biologically relevant microenvironment for new bone tissue development, improving the accuracy of preclinical models and clinical craniofacial repair outcomes.

#### 5.4.1. Stem Cell Applications for Bone Regeneration

**Figure 4.**
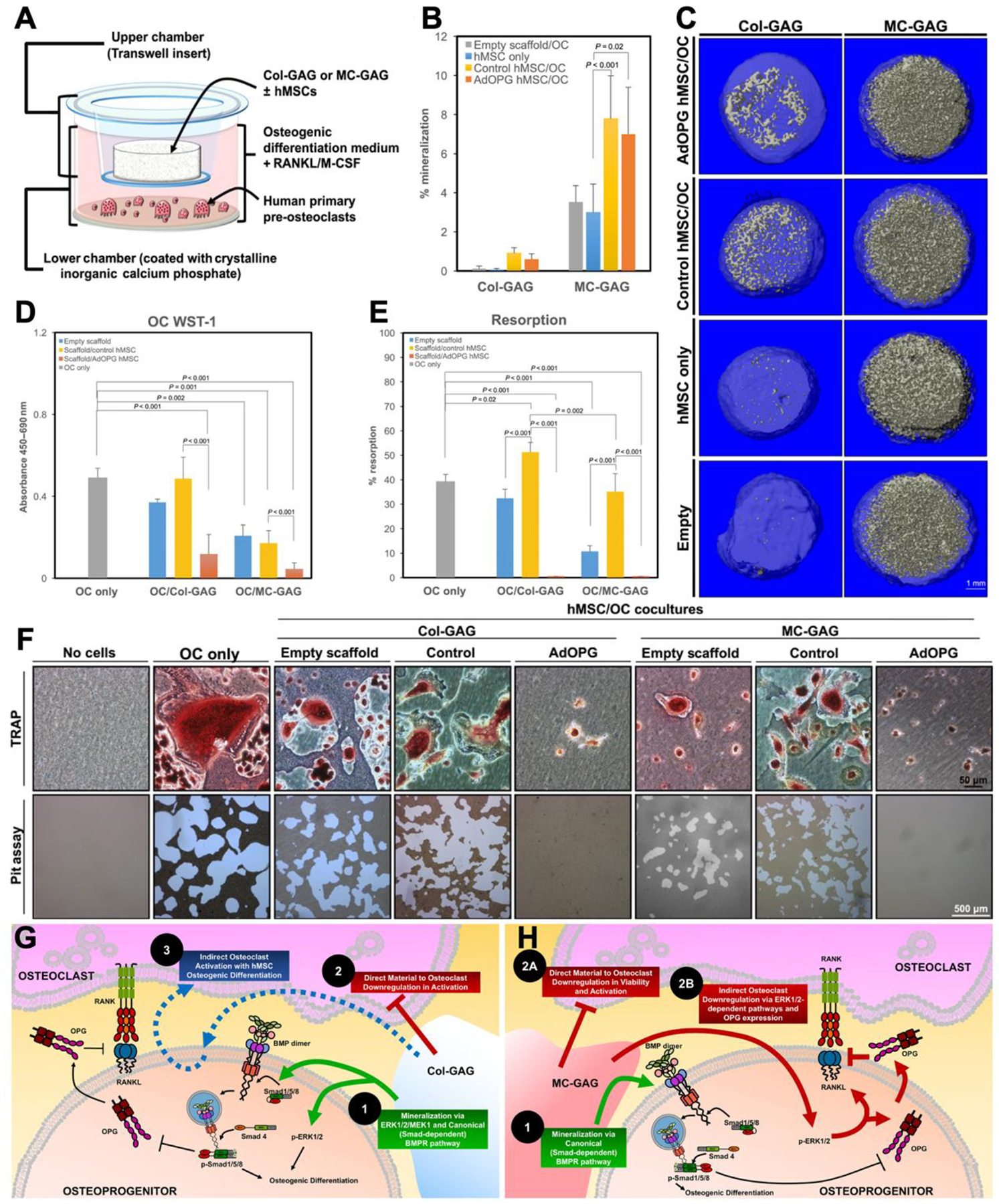
Mineralized collagen scaffolds modulate osteogenesis and osteoclast activity. Nanoparticulate mineralized collagen glycosaminoglycan (MC-GAG) scaffolds enhance human mesenchymal stem cells (hMSCs)-mediated bone formation and suppress osteoclast activity compared to nonmineralized collagen glycosaminoglycan (Col-GAG). (a) Schematic of the coculture system used to assess osteogenic and resorptive activity. (b-c) Quantification and microcomputed tomography (μCT) images show mineralization under different scaffold and cell conditions, including adenovirus-mediated delivery of osteoprotegerin (AdOPG)-transduced hMSCs. (d-f) MC-GAG scaffolds and AdOPG-transduced hMSCs reduce osteoclast proliferation and resorption as shown by WST-1 assays, pit assays, and TRAP staining. (g-h) Proposed mechanisms: Col-GAG supports osteogenesis but shows incomplete osteoclast inhibition, while MC-GAG more effectively promotes osteogenic differentiation and directly inhibits osteoclasts. Adapted from Ren et al., 2019.^[244,245]^

The most widely used cell type in regenerative bone engineering is MSCs, which are multipotent stromal cells that can differentiate into osteoblasts, chondrocytes, adipocytes, or myocytes.^[94]^ Unlike embryonic stem cells, MSCs are collected from adults in the bone marrow (BMMSCs), adipose tissue (ASCs), or umbilical cord (UCMSCs) and thus pose fewer ethical concerns. They are used for their immunosuppressive effects, and ability to secrete a wide range of regenerative growth factors and cytokines.^[40]^ Inducted pluripotent stem cells (iPSCs) have also been employed in bone regeneration.^[241,242]^ iPSCs are somatic cells, typically collected from blood or skin, which are then reprogrammed using transcription factors to a pluripotent state. Following this, they are selectively differentiated into MSCs, osteoclasts, and osteoblasts.^[243]^ Additionally, recent studies have demonstrated that nanoparticulate mineralized collagen scaffolds can modulate bone regeneration by reducing osteoclast resorption activity without impairing osteogenesis, while also directly and indirectly inhibiting osteoclastogenesis, highlighting their potential for improving MSC-based bone repair strategies (Figure 4).^[244,245]^

#### 5.4.2. Vascularization for Craniofacial Repair

Vascularization is critical for the survival and integration of engineered bone constructs, particularly in craniofacial bones, which endure substantial mechanical stress and strain.^[201,246]^ The integration of vascular networks not only supports cellular survival and function within scaffolds but also facilitates the necessary nutrient and oxygen supply for bone growth and remodeling. While 3D cell-laden scaffolds composed of synthetic polymers^[242]^, natural polymers^[247]^, and bioceramic-polymer composites^[248]^ have advanced craniofacial therapies, strategies to promote vascularization remain a major focus.^[152,246,249,250]^ Biomaterial design approaches, such as introducing controlled porosity, microchannels, and ECM-mimetic surfaces, enhance endothelial migration and vessel formation.^[249]^ Growth factor delivery, particularly the sustained release of VEGF, FGF-2, and PDGF through hydrogels or nanoparticle systems, has further improved neovascularization and bone healing outcomes.^[152,246,250]^

**Figure 5.**
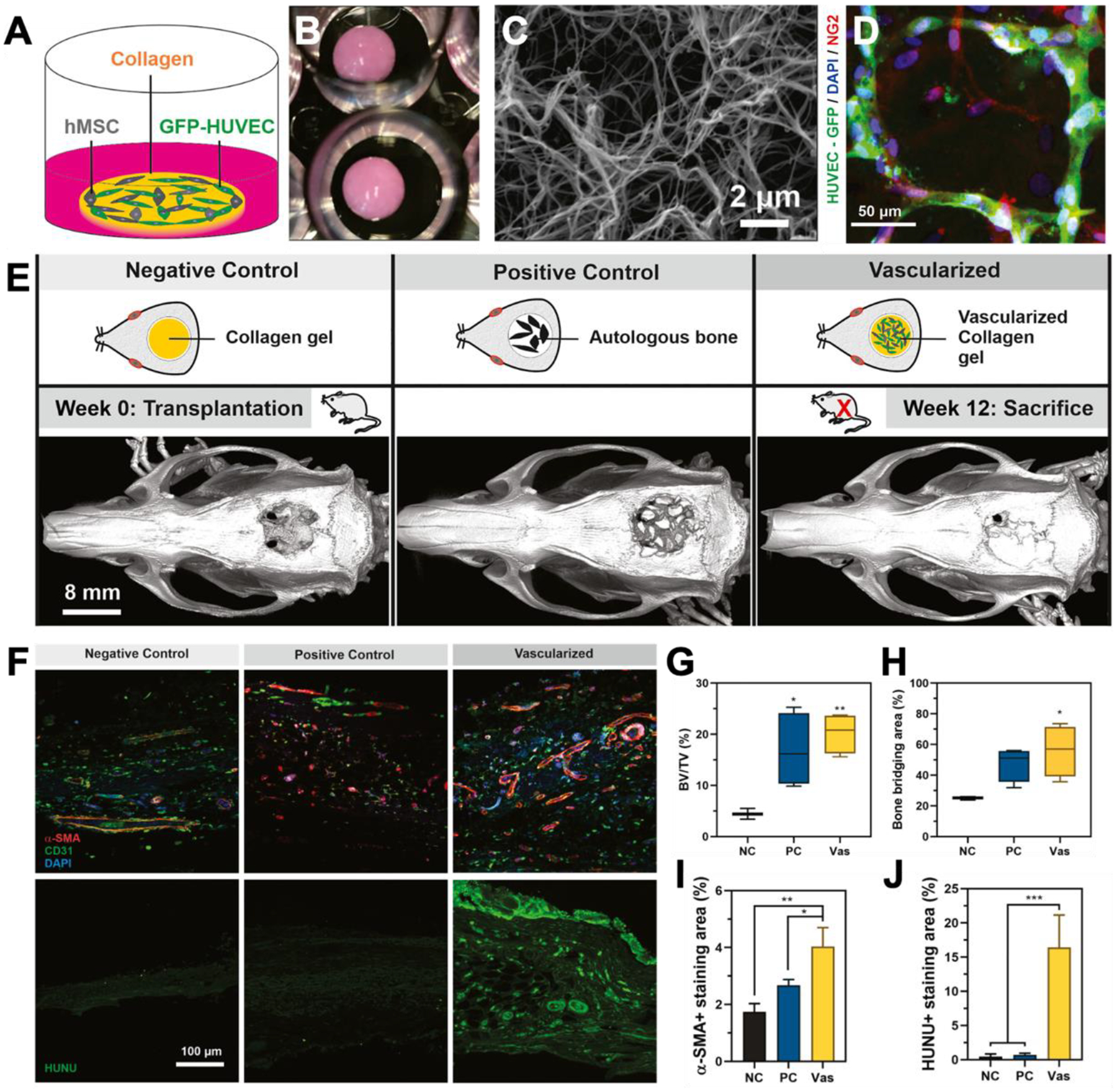
Pre-vascularized scaffolds enhance bone regeneration in a rat calvarial defect model. (a-d) Pre-vascularized scaffolds incorporating hBMSCs and HUVECs form mature, pericyte-supported vascular networks. (e) Schematic of in vivo implantation timeline and representative micro-CT images at 12 weeks. (f) Immunofluorescence staining of regenerated bone shows CD31, α-SMA, and HuNU expression, confirming a stable integration of human cells within the scaffold with the host’s vascular network. (g-j) Quantification reveals greater bone volume, bridging area, and vascular integration in mature pre-vascularized groups versus partially vascularized (PC) and non-vascularized controls (NC). Adapted from Subbiah et al., 2021.^[251]^

Additionally, co-culture methods combining mesenchymal stem cells (MSCs) with endothelial cells have enabled the engineering of pre-vascularized scaffolds with mature, pericyte-supported vascular networks, promoting integration with host vasculature and thereby the regeneration of critical-sized bone defects (Figure 5).^[251]^ Paracrine mechanisms from MSCs, including the secretion of pro-angiogenic factors and exosomes, have been shown to enhance endothelial cell migration and vascular network formation, further supporting coordinated bone and vascular regeneration.^[252–254]^ Although regenerative cell-laden scaffolds show promise for craniofacial regeneration, regulatory barriers continue to limit clinical translation.

### 5.5. Immunomodulation for Craniofacial Bone Regeneration

Immunomodulation has become a key strategy in craniofacial bone regeneration by managing the inflammatory response and facilitating tissue healing, vascularization, bone formation, and cell trafficking.^[255,256]^ Biomaterials modulate the immune response though surface chemistry, stiffness, topography, and degradation kinetics to promote immune cell infiltration, adhesion, signaling, or polarization. Promoting the transition from pro-inflammatory M1 to pro-healing M2 macrophages is critical for bone regeneration, as M2 cells secrete IL-10, recruit osteoprogenitor cells, support angiogenesis, and respond to biomaterial cues.^[257–269]^ Surface modifications, bioactive coatings, and incorporation of cytokines (e.g., IL-4, TGF-β) help shift the immune response from pro-inflammatory to pro-regenerative.^[256]^ MSC-membrane coatings and hydrophilic natural polymers like collagen and hyaluronic acid also support M2-dominant environments, improving integration and healing outcomes. Overall, tailoring biomaterial properties to modulate the immune response represents a critical strategy to enhance craniofacial bone regeneration, supporting both structural healing and functional restoration.

### 5.6. Strategies for Scaling Bone Regeneration

**Figure 6.**
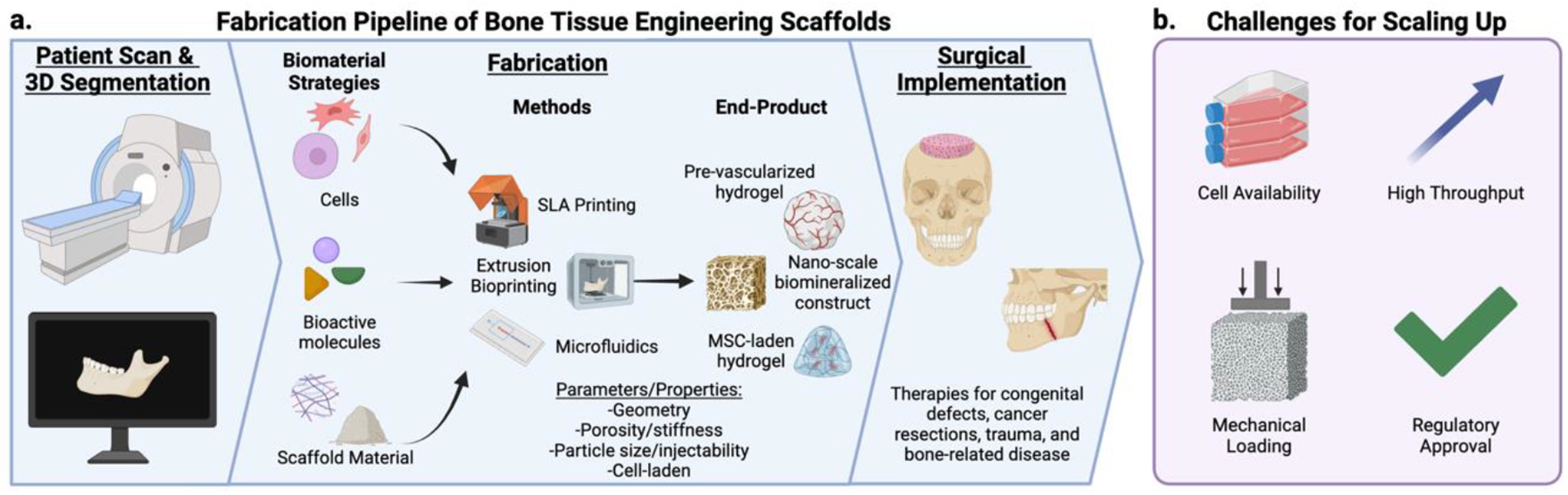
Fabrication pipeline for bone tissue engineering scaffolds and scalability challenges. (a) A typical fabrication pipeline starts with a patient scan to guide the fabrication process to follow. Different biomaterial strategies (e.g., cells, bioactive molecules, and scaffolding material) are employed with a fabrication method (e.g., 3D printing and microfluidics) to generate an end-product that can be used for surgical implantation. This pipeline can be used in therapies for common craniofacial reconstruction needs such as congenital defects, cancer resections, trauma, and bone-related diseases. (b) Main challenges to applying these strategies for scalable clinical use include cell availability, high-throughput production methods, mechanical loading of the scaffold, and regulatory approval. Created in BioRender.com.

Scaling engineered bone requires solutions that integrate efficacy, regulatory compliance, and clinical workflow compatibility (Figure 6). Established automated technologies, particularly additive manufacturing (AM) and microfluidics, are providing practical solutions for producing tissue engineered bone at scale. These systems also support the move toward personalized care by enabling patient-specific treatment strategies.

#### 5.6.1. Additive Manufacturing and Personalization

Commonly known as 3D printing, AM enables the rapid fabrication of complex structures using a wide array of materials—including bioceramics, synthetics and natural polymers, and metals.^[141,270]^ When integrated with 3D imaging, AM can be utilized to design and manufacture an implant that precisely matches a patient’s unique anatomical features, resulting in better implant fit and improved clinical outcomes.^[271–277]^ Modular scaffold designs – assembled from prefabricated building blocks - offer a hybrid approach, balancing personalization with surgical ease.^[278,279]^ Bioprinting, a subset of AM, represents a paradigm shift in tissue engineering by facilitating the inclusion of cells within scaffolds, which can lead to better integration and functionality regarding bone regeneration.^[175,270,280–284]^ In addition, bioprinting is continually advancing to support larger constructs with multiple materials, embedded cell types, and in clinically relevant geometries, moving closer to clinical translation.^[277,279–281,284]^ Many of these approaches utilize multiple materials via the inclusion of growth factors, cells, or ECM proteins directly in the bioink used for printing.^[141,277,279–281,283–286]^ Bioprinted constructs have a wide variety of applications in craniofacial tissue engineering, including bone defect repair, cranial reconstruction, and localized delivery of therapeutics.

#### 5.6.2. Microfluidics and Microparticles

**Figure 7.**
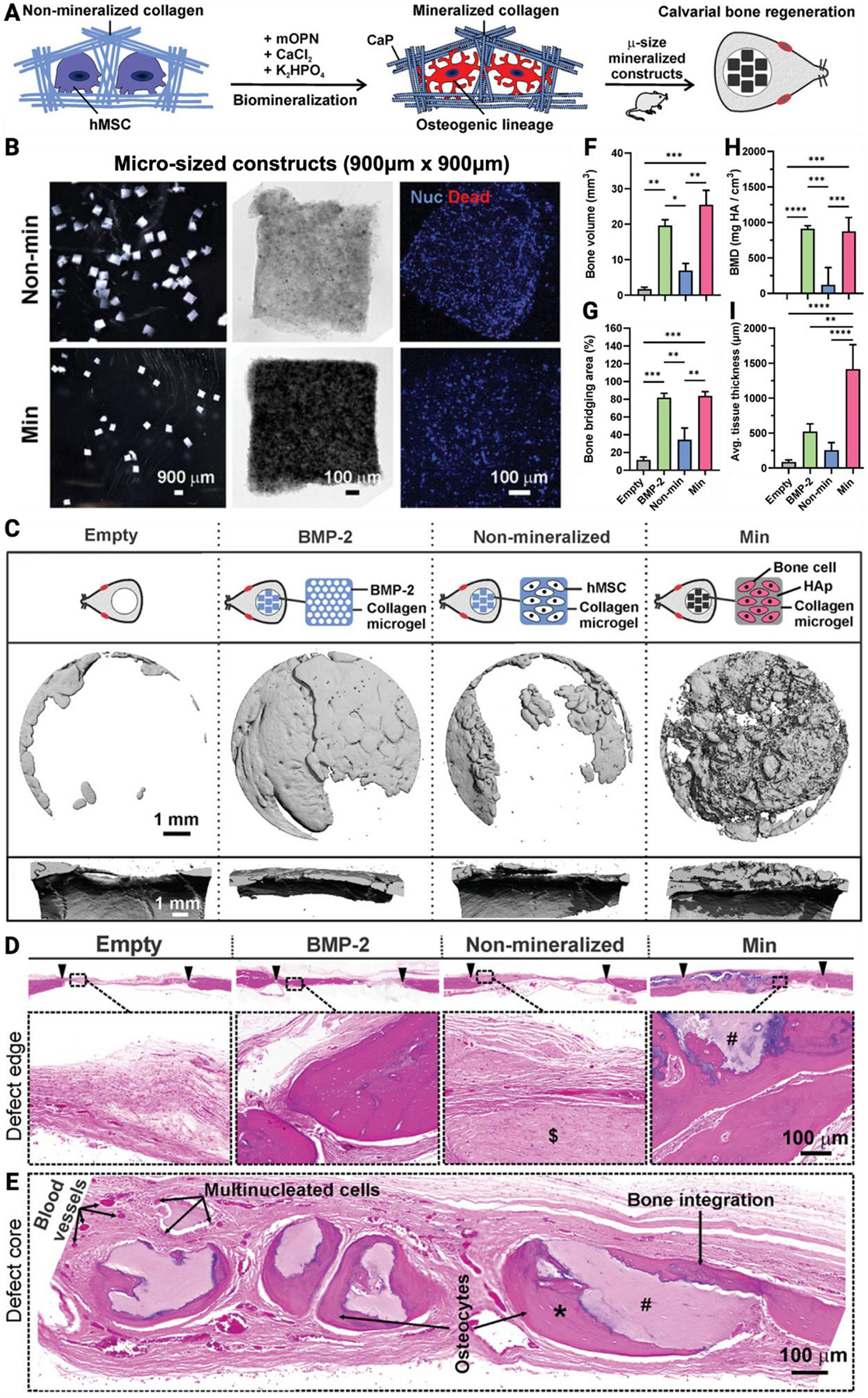
Injectable mineralized micro-constructs for bone regeneration. (a) Schematic of mineralized cell-laden collagen hydrogels. (b) Imaging and viability assay results at 72 hours. (c) Schematic of the 8 mm critical-sized calvarial defect model and treatment groups with representative micro-CT images at 12 weeks post-transplantation. (d) H&E-stained histological sections of bone tissue at 12 weeks, with arrowheads marking native versus regenerated bone. (e) High-magnification tile scan depicting mineralized construct integration into new bone at the defect core. (f-i) Quantitative micro-CT analysis demonstrating increased bone volume, bridging area, mineral density, and tissue thickness in mineralized groups. Adapted from Subbiah et al., 2023.^[288]^

Microfluidics enables high-throughput production of microparticles for use in bone tissue engineering. For small-scale craniofacial defects, injectable microparticles combined with cells, growth factors, or bioactive molecules have shown strong osteogenic potential (Figure 7).^[68,283,286–288]^ Nanoscale mineralization further improves scaffold strength, MSC differentiation, and osteoinductivity, especially when integrated into GelMA or collagen hydrogels.^[175,280]^ Among the available fabrication methods, microfluidic systems stand out for their precise control over particle size and internal architecture, critical parameters that influence the osteogenic potential of the microparticles.^[289,290]^ For example, scaffolds with a well-defined pore structure ranging from the nanoscale to the microscale have been shown to promote cell adhesion, support blood vessel formation, and facilitate complete healing of critical-sized bone defects within 12 weeks.^[290]^ Combining the structural integrity of large-scale AM constructs with microparticles has shown some promise in the literature and may be a promising approach for the clinical translation of craniofacial tissue engineering.^[278,279]^

Together, these innovations – including biomaterial tuning, stem cell engineering, vascularization strategies, immunomodulation, and scalable manufacturing – represent a multifaceted approach to overcoming key challenges in craniofacial bone regeneration and moving the field closer to clinical application. Scalable regenerative solutions will require convergence of fabrication technologies, regulatory alignment, and clinical implementation strategies to realize their full impact in patient care.

### 5.7. Strategies to Overcome Clinical Translation Barriers

Clinical translation of craniofacial tissue engineering strategies is limited by challenges such as scalability, cost, regulatory complexity, and variable outcomes. To address these challenges, several strategies have emerged that focus on optimizing biomaterial design, improving production efficiency, reducing costs, and validating safety and efficacy through clinical trials. Biomaterials must be engineered to balance biocompatibility, osteogenic capacity, feasibility of scalable fabrication, and implementation within surgical settings. Streamlined fabrication methods, such as 3D printing and microfluidics, can enhance production efficiency and lower manufacturing costs. Developing shelf-stable products, like lyophilized sterile scaffolds, may further easy surgical handling. Translational success also depends on advanced human organoid systems or rigorous preclinical studies using relevant animal models and well-designed clinical trials with standardized endpoints. Early collaboration with regulatory agencies can help align innovation with approval pathways and clinical implementation.

## 6. FUTURE DIRECTIONS

Several emerging innovations stand to transform craniofacial tissue engineering. One key advancement is bioprinting of multi-phasic vascularized tissues, enabling personalized implants that better replicate the anatomical and functional complexity of native craniofacial structures.^[291–293]^ These constructs must increasingly mimic physiological conditions, including flow dynamics, 3D architecture, and immune interactions. Rigorous validation will be essential to ensure they accurately represent human biology.

Patient-specific stem cell therapies also show promise for accelerating regeneration and minimizing immune rejection. By tailoring grafts to individual cellular and genetic profiles, these approaches enhance both integration and clinical outcomes. However, identifying causes of graft failure in specific populations – such as patients with aging-related deficits, chronic inflammation, or drug-resistant infections – remains a critical focus. Targeting immune pathways, such as macrophage polarization, offers a promising route to enhance healing in these settings.^[294]^ Additionally, the integration of bioengineered scaffolds with antimicrobial properties and immune-modulating capabilities represents a promising approach to addressing drug-resistant bone infections like osteomyelitis while simultaneously enhancing bone regeneration.

In parallel, bioengineered scaffolds with antimicrobial and immunomodulatory functions offer dual benefits: combating infections like osteomyelitis while promoting bone regeneration.^[295,296]^ Addressing the inflammatory imbalances of aging (e.g., “inflammaging”) through cytokine modulation or stem cell rejuvenation could further restore regenerative potential.^[297]^ By aligning with the patient's specific cellular, genetic, immunological, and pathological characteristics, these therapies ensure better integration, durability, and functionality of the graft, ultimately optimizing clinical outcomes.^[25]^

A major regulatory shift supporting innovation was the FDA Modernization Act 2.0 (2022), which expands the use of alternatives to animal testing, including organoids, organs-on-chips, and AI-based models.^[298]^ These tools may help accelerate translation while reducing reliance on models that poorly predict clinical outcomes.^[298]^ A major regulatory shift supporting innovation was the Food and Drug Administration (FDA) Modernization Act 2.0 (in 2022). This legislation expands the use of alternatives to traditional animal testing, including cell-based assays (e.g., human induced pluripotent stem cells, organoids, and organs-on-chips, and AI-based models) and advanced artificial intelligence approaches.^[298]^ These tools have the potential to streamline the preclinical pipeline, while reducing reliance on animal models that poorly predict clinical outcomes.^[298]^ Building on this milestone, in April 2025 the National Institutes of Health announced a new initiative to prioritize human-based research technologies. This initiative aims to expand the use of innovative, human-based research while reducing animal use. Developing and using cutting-edge alternative nonanimal research models aligns with the FDA’s recent initiative to also reduce testing in animals. In April 2025, the FDA announced a roadmap to phase out mandatory animal testing for several drug types. These include artificial intelligence-based models and engineered human organoids.

Nonetheless, cost and scalability remain a significant challenge. For instance, bioprinting complex tissues requires specialized equipment, high material volumes, and technical labor, while stem cell therapies involve resource-intensive workflows. These factors may limit widespread clinical adoption despite efficacy. To overcome these challenges, successful commercialization will depend on cross-sector collaboration among academia, industry, and regulatory bodies. Future efforts should prioritize the development of simpler, scalable, and lower-cost tissue models that maintain biological relevance while being broadly accessible. Democratizing these tools can extend beyond craniofacial repair to other areas of regenerative medicine. As the field advances, future directions will rely on the integration of personalized stem cell therapies, multifunctional biomaterials, and validated preclinical models to overcome translational barriers and improve regenerative outcomes in complex craniofacial defects.

## 7. CONCLUSION

Craniofacial tissue engineering holds tremendous promise for restoring function and aesthetics in patients with large bone defects. Although progress has been made, replicating the mechanical, biological, and physiological properties of native bone remains a major challenge. Advances in nanoscale scaffold design, stem cell-based strategies, and bioprinting are beginning to address these barriers.

Harnessing the biochemical, structural, and mechanical cues of the extracellular matrix can guide cell differentiation and function, offering a pathway to engineer grafts with native-like behavior. Mechanobiology is emerging as a critical focus area, especially in mechanically dynamic environments like the craniofacial complex.

However, clinical translational will require addressing persistent barriers, including cost, scalability, and regulatory complexity. By fostering interdisciplinary collaboration and prioritizing strategies that align with both biological fidelity and clinical practicality, the field is poised to deliver personalized, sustainable solutions for craniofacial regeneration and beyond.

## Figures and Tables

**Table 1.** Advantages, disadvantages, and clinical availability of common biomaterials used for craniofacial bone regeneration scaffolds

Biomaterial Category	Biomaterial	Advantages	Disadvantages	Source
*Bioceramics*	Calcium Phosphates**	Similar chemical composition to native bone’s mineral phase Excellent osteoinductivity Excellent absorbability Injectable Degradation products of calcium and phosphate are bioresorbable	Brittleness limits load-bearing capabilities Degradation rate may be slower than new bone formation	^[83]^ ^[84]^ ^[85]^ ^[86]^
	Calcium Silicates**	Silicon ions from degradation facilitate osteoconductivity and angiogenesis Accelerates apatite deposition	Poor handling characteristics Long setting time Fast degradation	^[83]^ ^[87]^ ^[88]^ ^[89]^
	Bioactive Glass**	Osteoconductive Bioactive, activates osteogenic genes Alkaline nature allows for antibacterial property Degradation mechanism forms a HA layer on scaffold surface and neighboring native tissue	Intrinsic brittleness Low fracture toughness	^[83]^ ^[90]^ ^[91]^ ^[92]^ ^[93]^
*Synthetic Polymers*	Polylactic acid (PLA)	Biodegradable Strong mechanical properties Scalable manufacture Compatible with 3D printing techniques for customized scaffold	Lactic acid degradation product can create acidic environment and cause inflammation Prone to hydrolysis Hydrophobic nature discourages cell adhesion	^[94]^ ^[95]^ ^[96]^
	Polycaprolactone (PCL)	Flexible material compared to other biomaterial polyesters High mechanical strength Biodegradable Shape memory fabrication	Hydrophobic nature discourages cell adhesion High transition temperature needed for shape actuation Slowest degradation rate amongst biomaterial polyesters (3–4 years) 6-hydroxycaproic acid degradation product can create acidic environment and cause inflammation	^[97]^ ^[98]^ ^[99]^ ^[94]^ ^[100]^
	Polyglycolic acid (PGA)	Biodegradable Controllable degradation rate	Poor biocompatibility and cellular adhesion Glycolic acid degradation product can create acidic environment and cause inflammation	^[101]^ ^[102]^ ^[103]^ ^[104]^
	Polyethylene glycol (PEG)**	Hydrophilic Bioinert	Limited mechanical strength Not biodegradable, requires chemical modification	^[83]^ ^[105]^ ^[106]^
	Poly (lactide-co-glycolide) (PLGA)**	Porosity control Tunable degradation rate	Poor osteoconductivity Suboptimal mechanical strength due to amorphous structure Glycolic and lactic acid degradation products can create acidic environment, and acidic breakdown can cause inflammation	^[97]^ ^[107]^ ^[108]^
*Natural Polymers*	Collagen**	Naturally occurring in bone tissue, osteoconductive Biodegradable, degradation products are bioresorbable Strong plasticity Easy absorption of growth factors, minerals, molecules	Poor mechanical strength Risk of immunogenicity if derived from animal sources	^[83]^ ^[109]^ ^[104]^
	Demineralized Bone Matrix**	Enables rapid revascularization Natural bone matrix constituents arranged in native structure Retains native growth factors Biodegradable, degradation products are bioresorbable	Osteoinductive variability based on allograft donors, processing conditions, sterilization techniques, and handling methods Potential risk of virus transmission	^[110]^ ^[111]^ ^[112]^
	Gelatin	Biocompatible Low toxicity Low allergenicity Biodegradable, degradation products of collagen fragments and peptides are bioresorbable	Weak mechanical properties Lacks thermal stability	^[103]^ ^[113]^ ^[114]^
	Chitosan	Cationic nature allows for interactions with glycosaminoglycans (GAGs) and proteoglycans, which modulate the bone microenvironment Antibacterial properties Anti-inflammatory Biodegradable, degradation products of amino sugars and saccharides are bioresorbable	Fast biodegradation Poor osteoconductivity	^[115]^ ^[116]^ ^[117]^ ^[118]^
	Silk	Strong tensile strength Osteoconductive Flexible Degradation rate can be tailored Biodegradation facilitated by macrophages, osteoblasts, and osteoclasts, products are bioresorbable	High brittleness and fragility	^[119]^ ^[103]^ ^[108]^ ^[120]^
	Alginate**	Relatively low cost Easily chemically modified Gel-forming ability	Sterilization procedure causes degradation Not biodegradable, must be chemically modified	^[107]^ ^[108]^ ^[121]^ ^[104]^
*Metals*	Titanium & Titanium Alloys**	Greater corrosion resistance Superior biocompatibility Relatively lower weight and density	Not biodegradable Higher cost to manufacture Corrosion byproducts can be cytotoxic	^[122]^ ^[123]^
	Stainless Steel	Inexpensive and readily available Mechanical strength	Higher density metal Low corrosion resistance Not biodegradable	^[124]^
	Chromium-Cobalt**	Biocompatibility Mechanical strength	Expensive to manufacture Not biodegradable	^[122]^ ^[125]^ ^[126]^
	Zinc & Zinc Alloys**	Degradation rate is comparable to native bone growth Biodegradation product of zinc is bioresorbable	Poor strength and ductility	^[127]^ ^[122]^ ^[128]^
	Iron & Iron Alloys	Mechanical strength Biodegradation product of iron is bioresorbable	Slow degradation rate relative to physiological bone remodeling	^[122]^
	Magnesium & Magnesium Alloys**	Similar ductility and density to natural bone Degradation byproduct of magnesium is bioresorbable	Degradation rate is too fast in physiological environments	^[129]^ ^[130]^ ^[128]^ ^[131]^ ^[131]^

** biomaterial available on the market for clinical craniofacial regeneration purposes
